# New hypoglycemic agents and the kidney: what do the major trials tell us?

**DOI:** 10.12688/f1000research.16135.1

**Published:** 2018-11-23

**Authors:** Brendan Smyth, Vlado Perkovic

**Affiliations:** 1 The George Institute for Global Health, UNSW, Sydney, Australia

**Keywords:** Chronic Kidney Disease, Diabetic nephropathy, macroalbuminuria, empagliflozin, canagliflozin, SGLT2, GLP-1, liraglutide, semaglutide, saxagliptin, alogliptin, DPP-4

## Abstract

As the burden of diabetic kidney disease continues to expand, new therapies to preserve renal function or prevent diabetic nephropathy are urgently needed. In the past decade, a number of new hypoglycemic classes have emerged, each with a unique profile of action and benefits. Here we review the impact of glycemic control on renal outcomes and the results of the major clinical trials of glucagon-like peptide 1 (GLP-1) agonists, dipeptidyl peptidase-4 (DPP-4) inhibitors, and sodium–glucose co-transporter 2 (SGLT2) inhibitors. Both GLP-1 agonists and SGLT2 inhibitors consistently demonstrate renal benefits. Further studies of these new agents in different patient groups and in comparison to (or in combination with) other treatments are required to better define their role in combating the burden of diabetic kidney disease.

## Introduction

In 2016, chronic kidney disease (CKD) due to diabetes mellitus (DM) was responsible for the loss of almost 15 million disability-adjusted life years worldwide, an increase of 25% over the preceding 10 years ^1^. Despite important advances in therapy, it still constitutes a major challenge to patients, clinicians, and healthcare services and results in dramatically shortened lifespan, lower quality of life, and increased healthcare costs ^2^. Prevention of advanced diabetic nephropathy by either preventing disease onset or slowing the decline of established CKD is a critical goal of therapy. Angiotensin system blockade is well established as an effective treatment for albuminuria in diabetic nephropathy and is known to slow disease progression ^3^. Multiple novel agents have been investigated to date but have frequently proven less effective or less well tolerated than current therapies ^4^. As such, glucose control remains the primary therapy in patients with diabetes. The effect of specific classes of hypoglycemic agents on renal outcomes is therefore a critical consideration in the management of diabetes and diabetic nephropathy. In the past decade, three new classes of hypoglycemic agent have entered the market: glucagon-like peptide 1 (GLP-1) receptor agonists, dipeptidyl peptidase-4 (DPP-4) inhibitors, and sodium–glucose co-transporter 2 (SGLT2) inhibitors. After reviewing the impact of tight glycemic control on renal disease, this review will focus on the renal outcomes in major trials of these new agents in patients with type 2 DM (T2DM).

## Glycemic control and diabetic nephropathy

Our understanding of the impact of glycemic control on diabetic microvascular outcomes in T2DM is derived primarily from a series of large randomized controlled trials (RCTs) of varying glycemic targets. The United Kingdom Prospective Diabetes Study (UKPDS), a watershed in the management of T2DM, demonstrated a reduction in microvascular outcomes (primarily due to a lower rate of retinopathy) with a target fasting glucose of <6 mmol/L versus the conventional target of <15 mmol/L (resulting in a mean HbA1c of 7.0% versus 7.9%, respectively) ^5^. This was followed a decade later by a cluster of trials––ADVANCE ^6^, ACCORD ^7^, and VADT ^8^––aiming to determine the optimal HbA1c target and enrolling a cohort of generally older participants with a median time since diagnosis of diabetes of 7–10 years, many of whom had established microvascular and/or macrovascular disease. Collectively, these four trials enrolled 27,049 participants with a median follow up of 5.0 years. A recent meta-analysis using individual patient data provides the highest quality evidence for the impact of tighter glycemic control on renal outcomes ^9^. The mean difference in HbA1c in the more-intensive versus less-intensive arms was approximately 1% (HbA1c 6.80% [95% confidence interval (CI) 6.65, 6.95] versus 7.74% [95% CI 7.34, 8.14], respectively). This was associated with a 20% reduction (hazard ratio [HR] 0.80 [95% CI 0.72, 0.88]) in the composite of end-stage kidney disease (ESKD), renal death, estimated glomerular filtration rate (eGFR) of <30 mL/minute/1.73 m ^2^, and new macroalbuminuria ^9^. This outcome occurred in 1.2% of the more-intensive arm and 1.6% of the less-intensive arm and was primarily driven by a reduction in the rate of transformation from normoalbuminuria (<30 mg/g or <3 mg/mmol) or microalbuminuria (30–300 mg/g or 3–30 mg/mmol) to overt diabetic nephropathy with macroalbuminuria (>300 mg/g or >30 mg/mmol). Interestingly, the risk of decline in eGFR to <30 mL/minute/1.73 m ^2^ was not affected by tighter glycemic control (HR 1.16 [95% CI 0.93, 1.44]), and, while the ADVANCE trial found a reduced risk of ESKD in participants randomized to tight control ^10^, this was not seen across the other trials ^9^. The meta-analysis identified an increased risk of severe hypoglycemia (HR 2.48 [95% CI 1.91, 3.21]) in those treated with intensive glucose lowering and, despite a reduction in major cardiovascular events (HR 0.91 [95% CI 0.84, 0.99]), there was no reduction in all-cause mortality (HR 1.04 [95% CI 0.90, 1.20]) ^11^. Indeed, in the ACCORD study, intensive glucose lowering (achieved median HbA1c of 6.4%) was associated with an increase in mortality (HR 1.22 [95% CI 1.01, 1.46]; *p*=0.04) ^7^. While these trials and their subsequent analysis confirm that further modest reductions in renal events are achievable with more-intensive glycemic control, they also highlight the increasing risks and diminishing returns of this approach. These findings suggest the need for further therapies capable of preventing the loss of kidney function independently of reductions in glycemia.

## Incretin mimetics and enhancers

Two of the three new classes of hypoglycemic agents, GLP-1 receptor agonists and DPP-4 inhibitors, act by augmenting incretins, a family of gut-derived hormones that potentiate the secretion of insulin while also exerting a variety of potentially beneficial metabolic effects independent of insulin activity, including enhanced satiety, reduced gastric emptying, and intestinal motility, which may lead to reductions in body weight and waist circumference ^12^. GLP-1 is the most important of the incretin hormones from a therapeutic perspective, as its action can now be modulated either by direct agonism or by inhibition of the enzyme primarily responsible for its breakdown (DPP-4) ^12^.

### Glucagon-like peptide 1 receptor agonists

GLP-1 receptor agonists are peptides administered subcutaneously with substantial homology with endogenous GLP-1 but which are resistant to metabolism by DPP-4. Owing to partial renal clearance, GLP-1 receptor agonists were initially restricted to patients with an eGFR of >30 mL/minute/1.73 m ^2^ ^12^; however, following further regulatory review, they are now approved for CKD stage 4 in some regions.

Four major RCTs have examined the cardiovascular effects of GLP-1 receptor agonists in participants with T2DM and a history or risk of cardiovascular disease: ELIXA (lixisenatide, n=6,068) ^13^, LEADER (liraglutide, n=9,340) ^14^, SUSTAIN-6 (semaglutide, n=3,297) ^15^, and EXSCEL (extended-release exenatide, n=14,752) ^16^. Meta-analysis of these studies has demonstrated a reduction in risk of all-cause mortality (HR 0.88 [95% CI 0.81, 0.95]), cardiovascular mortality (HR 0.87 [95% CI 0.79, 0.96]), and major adverse cardiovascular events (HR 0.90 [95% CI 0.82, 0.99]) ^17^. Favorable reductions in HbA1c (–0.57% [95% CI –0.74, –0.40]), body weight (–2.25 kg [95% CI –3.09, –1.41]), and systolic blood pressure (–1.33 mmHg [95% CI –1.80, –0.86]) were also reported in a separate meta-analysis ^18^. In addition to these metabolic benefits, previous studies suggest that GLP-1 receptor agonists have complex intra-renal effects, including natriuresis and afferent arteriolar vasodilation ^12^. The extent to which the renal hemodynamic effects of GLP-1 receptor agonists affect renal outcomes is not clear. These renal actions of GLP-1 appear complex, with renal afferent arteriolar vasodilation and glomerular hyperfiltration demonstrated in healthy individuals but not in those with T2DM, in whom a reduction in glomerular hyperfiltration or no change has been reported. Subsequent trials have generally not shown acute changes in eGFR when commencing GLP-1 receptor agonists.

Three of the four large GLP-1 agonist RCTs have reported renal outcomes. In the LEADER trial, with a reduction in HbA1c of approximately 1.0% in the first 12 months (decreasing to 0.4% at 36 months) and of 2.5 kg in bodyweight, the composite renal outcome (new macroalbuminuria, doubling of creatinine, eGFR of ≤45 mL/minute/1.73 m ^2^, and renal replacement therapy [RRT] or renal death) was reduced significantly in the liraglutide arm (HR 0.78 [95% CI 0.67, 0.92]) driven by a reduction in macroalbuminuria (9.0% versus 12.1%) ^14^. A reduction in the rate of decline in renal function was also noted in post-hoc analyses ^19^. Overall, the rate of decline was 2% lower in the liraglutide group (a difference of doubtful clinical importance); however, in those with impaired renal function at baseline (eGFR 30–59 mL/minute/1.73 m ^2^), the rate of decline was 2 mL/minute/1.73 m ^2^ per year in the liraglutide group as compared to 4 mL/minute/1.73 m ^2^ per year in the placebo group ( *p*<0.001). No effect was observed on the risk of ESKD (HR 0.87 [95% CI 0.61, 1.24]). The results of SUSTAIN-6 were similar, with a similar renal composite endpoint (not including renal death) being less frequent in the treatment group (HR 0.64 [95% CI 0.46, 0.88]) ^15^, although it is not known if rate of decline in eGFR was affected by semaglutide, as this analysis has not been published to date. The ELIXA trial demonstrated that lixisenatide was associated with a lower risk of new-onset macroalbuminuria (HR 0.81 [95% CI 0.66, 0.99]), but there was no difference in decline in eGFR or doubling of serum creatinine (although the number of events was small) ^20^. Finally, a recent randomized study in 577 participants with stage 3–4 CKD and T2DM showed that glycemic control and safety of dulaglutide were similar to insulin glargine. Patients treated with dulaglutide sustained approximately 2 kg of weight loss and had lower rates of hypoglycemia. There was a significant difference between decline in cystatin C-eGFR in the dulaglutide arm and the insulin arm over 12 months (–0.7 mL/minute/1.73 m ^2^ versus –3.3 mL/minute/1.73 m ^2^; *p*<0.05), but this did not reach significance when measured by creatinine-eGFR ^21^. It remains unclear whether dulaglutide affects the underlying rate of decline in eGFR or the development of ESKD.

### Dipeptidyl peptidase 4 (DPP-4) inhibitors

Our understanding of the renal effects of DPP-4 inhibitors is derived from four large placebo-controlled RCTs ^12^. These trials have shown these agents to be safe in patients with CKD, but the benefits appear to be limited to modest improvements in albuminuria. Moreover, no reductions in major cardiovascular events were demonstrated ^22– 25^. In the 16,492 participants in the SAVOR-TIMI 53 trial of saxagliptin versus placebo ^25^, there was a significant difference in the number with stable or improved albuminuria category in favor of the saxagliptin arm (after a median follow up of 2.1 years). At the 2-year follow up point, mean albumin creatinine ratio was lower by 34.3 mg/g (3.88 mg/mmol) ( *p*<0.004). The reduction in albuminuria was most marked in participants with an eGFR of <30 mL/minute/1.73 m ^2^, although this did not reach significance: 245.2 mg/g (27.7 mg/mmol) ( *p*=0.086). No differences in change in eGFR, doubling of serum creatinine, or ESKD were identified ^26^. Albuminuria was not studied in the EXAMINE trial of alogliptin after acute coronary syndrome (n=5,380), but, as with saxagliptin, no significant changes in eGFR or ESKD were identified ^24^. The TECOS trial of sitagliptin versus placebo (n=14,671) also did not demonstrate a difference in rate of decline of renal function, irrespective of eGFR at baseline ^27^. A trivial difference in urine albumin creatinine ratio was identified (–0.18 mg/g [95% CI –0.35, –0.02]), but there was no difference in incident microalbuminuria or ESKD, even in those with baseline CKD ^27, 28^. The most recent of these studies, the CARMELINA trial (n=6,979) ^25^, which enrolled participants with both high cardiovascular risk (a history of vascular disease) and renal risk (reduced eGFR and microalbuminuria or macroalbuminuria), found no difference in rates of the composite renal outcome of ≥40% reduction in eGFR, ESKD, or death from renal failure (HR 1.04 [95% CI 0.89, 1.22]). This was despite a significant reduction in progression of albuminuria (HR 0.86 [95% CI 0.78, 0.95]). Overall, it appears that the impact of DPP-4 inhibitors on renal outcomes is modest at best, a finding supported by a meta-analysis of 36 DPP-4 inhibitor RCTs (excluding CARMELINA) which concluded that there was no impact on the risk of renal failure (RR 1.06 [95% CI 0.88, 1.27]) ^29^.

## Sodium–glucose co-transporter 2 inhibitors

SGLT2 inhibitors, a relatively new class of hypoglycemic agent, have demonstrated both cardiovascular and renal benefits ^30^. Inhibition of SGLT2 results in lowered serum glucose levels via an increase in urinary glucose excretion. This novel mechanism of action also results in an increase in sodium delivery to the distal tubule, triggering tubuloglomerular feedback (via the macula densa) and a reduction in glomerular pressure. A study in 40 participants with T1DM suggests that this mechanism results in reduced renal blood flow and glomerular filtration, particularly in those with baseline hyperfiltration ^31^. As with angiotensin blockade, this results in a reduction in proteinuria. The net effect of this is a 3–5 mL/minute/1.73 m ^2^ reduction in eGFR and a reduction in albuminuria, independent of lowering of HbA1c. In addition, the diuretic effect of increased glucose delivery to the distal tubule lowers blood volume and blood pressure and the loss of glucose represents a net loss of calories, contributing to weight loss.

Two major clinical trials of SGLT2 inhibitors have been published, with several more in progress ^32^. The EMPA-REG OUTCOME study of empagliflozin enrolled 7,020 participants with established cardiovascular disease, T2DM, and an eGFR of >30 mL/minute/1.73 m ^2^ who were followed for a median of 3.1 years ^33^. The primary outcome of cardiovascular death, myocardial infarction, and stroke was reduced by 14% (HR 0.86 [95% CI 0.74, 0.99]), and there was a 35% reduction in hospitalization for heart failure (HR 0.65 [95% CI 0.50, 0.85]). The composite renal outcome of incident macroalbuminuria, doubling of serum creatinine, eGFR of <45 mL/minute/1.73 m ^2^, and RRT or death from renal disease occurred in 12.7% of those assigned to empagliflozin and 18.8% of those in the placebo arm (HR 0.61 [95% CI 0.53, 0.70]) ^34^. This benefit was consistent among subgroups and dose of empagliflozin. Although largely driven by a reduction in progression to macroalbuminuria (11.2% versus 16.2%), the other components of the composite outcome were also significantly reduced by similar relative magnitudes: doubling of serum creatinine (1.5% versus 2.6%) and need for RRT (0.3% versus 0.6%). Empagliflozin also resulted in a significantly lower rate of decline in eGFR: 0.19±0.11 mL/minute/1.73 m ^2^ per year versus 1.67±0.13 mL/minute/1.73 m ^2^ per year (after adjusting for the 4–5 mL/minute/1.73 m ^2^ initial decline in eGFR seen in the initial month of treatment, which was reversible upon trial completion) ^34^.

The CANVAS Program comprised two RCTs comparing canagliflozin to placebo in 10,142 participants with T2DM at high cardiovascular risk (i.e. previous symptomatic coronary artery disease or age over 50 with two risk factors). Participants were followed for a mean of 3.6 years. Like EMPA-REG OUTCOME, the CANVAS trial saw a decrease in major cardiovascular events, heart failure, and composite renal endpoint of sustained doubling of serum creatinine, ESKD, or death from renal causes, which occurred at a rate of 1.5 versus 2.8 per 1,000 patient-years (HR 0.53 [95% CI 0.33, 0.84]) ^35^. There was a 42% reduction in incident macroalbuminuria (HR 0.58 [95% CI 0.50, 0.68]) and a 50% reduction in doubling of serum creatinine (HR 0.50 [95% CI 0.30, 0.84]). There was no significant reduction in ESKD or renal death; however, only 21 events were recorded overall, and the point estimate of effect was consistent with the broader composite outcome (HR 0.56 [95% CI 0.23, 1.32]). Renal function was also stabilized in those on canagliflozin (once the initial 3.1 mL/minute/1.73 m ^2^ decrease in eGFR was accounted for), with a mean annual change in eGFR of +0.3 mL/minute/1.73 m ^2^ versus –0.9 mL/minute/1.73 m ^2^ in those on placebo. This resulted in a mean difference in annual eGFR decline of 1.2 mL/minute/1.73 m ^2^ per year (95% CI 1.0, 1.4) ^36^.

The most common side effect of SGLT2 inhibitors is fungal genital infections, which affected approximately 5% of male patients and 10% of female patients in both trials ^33, 35^. There were no increases in bacterial urinary tract infections, including complicated urinary tract infections, nor in the incidence of acute kidney injury. Based on earlier studies, the U.S. Food and Drug Administration (FDA) inserted a label warning regarding acute kidney injury for SGLT2 inhibitors. While cautious initiation (as with any diuretic) is reasonable, the favorable long-term renal effects in both CANVAS and EMPA-REG OUTCOME suggest that any such acute, volume-related renal injury rarely leads to permanent loss of renal function ^37^. An unexpected increase in the rate of amputations (predominantly toe or metatarsal) was noted with canagliflozin (6.3 versus 3.4 per 1,000 patient-years; HR 1.97 [95% CI 1.41, 2.75]) ^35^. While this has not been noted in the only other completed trial of SGLT2 inhibitors (EMPA-REG OUTCOME), a numerical excess has been observed in the ertugliflozin development program ^38^. It is thus unclear whether this is a drug effect, class effect, or chance finding ^30^. Observational studies of these associations have not been consistent ^30^. Concern has also been raised regarding the increased risk of fractures seen with canagliflozin, including in the CANVAS trial. A meta-analysis identified a 22% increased risk of fractures with canagliflozin (HR 1.22 [95% CI 1.02, 1.46]), but no significant differences for other agents in the SGLT2 class ^37^, again raising the possibility that this is a drug effect rather than a property of the class. In addition, post-marketing reports to the FDA have highlighted 12 cases of Fournier’s gangrene (necrotizing fasciitis of the perineum), prompting a specific label warning. Finally, cases of diabetic ketoacidosis (DKA) have been reported with SGLT2 inhibitors, mostly in T1DM patients, and, while these appear to be rare in patients with T2DM, it is important that clinicians are aware that patients may present with only mildly elevated blood glucose levels. Reassuringly, there was no significant increase in DKA seen in either the EMPA-REG OUTCOME or the CANVAS trial ^33, 35^.

## Conclusion

To conclude, the range of options for the treatment of T2DM continues to expand. Of the three new classes of hypoglycemic agents released in the past decade, while DPP-4 inhibitors do not appear to have obvious renal benefits, both GLP-1 receptor agonists and SGLT2 inhibitors are now proven to reduce the risk of progression to macroalbuminuria. More importantly, SGLT2 inhibitors also consistently reduce the incidence of progression of CKD, whether measured by doubling of serum creatinine or rate of decline in eGFR. The physiology of these agents, the results of the trials above, and the adverse event profiles suggest that some drug classes may be better suited to particular patient subpopulations and, as yet, these benefits cannot be extrapolated to patients with an eGFR of <30 mL/minute/1.73m ^2^, an important group for whom the risks of progressive loss of renal function are highest. Further studies of these new agents in different patient groups and in comparison to (or in combination with) other treatments are required to better define their role in combating the burden of diabetic kidney disease.
